# Permeation-enhancing effects and mechanisms of *O*-acylterpineol on isosorbide dinitrate: mechanistic insights based on ATR-FTIR spectroscopy, molecular modeling, and CLSM images

**DOI:** 10.1080/10717544.2018.1561764

**Published:** 2019-02-11

**Authors:** Yan Li, Chunyan Wang, Jian Wang, Tianzhe Chu, Linlin Zhao, Ligang Zhao

**Affiliations:** a School of Pharmacy, North China University of Science and Technology, Tangshan, China;; b Department of Pharmacy, Tangshan Maternal and Child Health Hospital, Tangshan, China;; c Department of Pharmaceutical Sciences, Shenyang Pharmaceutical University, Shenyang, China;; d Tangshan Key Laboratory of Novel Preparations and Drug Release Technology, Tangshan, China

**Keywords:** *O*-acylterpineol derivatives, penetration enhancers, fatty acid, transdermal drug delivery, penetraion mechanism

## Abstract

The present study aimed to evaluate the penetration activity of *O*-acylterpineol derivatives both *in vitro* and *in vivo*, and to investigate the enhancing mechanism of *O*-acylterpineol derivatives which were synthesized by α-terpineol and fatty acid. The promoting activities on the isosorbide dinitrate patch were tested across full thickness rabbit skin both *in vitro* and *in vivo*. In order to elucidate the permeation mechanism, attenuated total reflection Fourier transform infrared spectroscopy, molecular modeling, and confocal laser scanning microscopy were introduced to investigate the regulation of enhancers in the skin permeability and biophysical properties. With *in vitro* cytotoxicity test and *in vivo* erythema model, the skin irritation of enhancers was also evaluated. Permeation studies showed 2-(4-methylcyclohex-3-en-l-yl) propan-2-yl tetradecanoate produced the obvious enhancement activity for ISDN both *in vitro* and *in vivo* from patches. These results were supported by ATR-FTIR, molecular modeling, and CLSM studies which revealed that *O*-acylterpineol could decrease the order of the alkyl chains in the skin lipids. Additionally, it was found that TER-C14 produced a relatively low skin irritation, compared with the TER which was assumed to be a safe compound. The present research suggested that some newly designed acylterpineol derivatives are shown to be suitable permeation enhancers for transdermal drug delivery, and the chain length of C14 seem to be safe and more favorable for the penetration of ISDN from DIA patches.

## Introduction

For decades, transdermal drug delivery has been accepted as a potential noninvasive route of administration, with advantages of prolonged therapeutic action, decreased side effect, easy use, and better patient compliance. However, the major limitation for transdermal drug delivery system (TDDS) is the skin itself. The barrier function of the stratum corneum (SC) is attributed to its multilayered wall-like structure, in which terminally differentiated keratin-rich epidermal cells (corneocytes) are embedded in an intercellular lipid-rich matrix (Hui et al., 2005). Consequently, to achieve reasonable bioavailability, hydrophilic or lipophilic penetration enhancers which reduce the skin barrier function by a reversible decrease of the SC lipid order, are widely used (Hadgraft, 1999; Williams & Barry, 2004). To date, hundreds of substances are synthesized to enhance drug permeation across the skin (Marwah et al., 2016; Kadhum et al., 2017; Li et al., 2018; Dai et al., 2018). However, as a result of their incompatibility in the formulation or local irritation issues, few to date have been routinely incorporated in the currently marketed transdermal products. Terpenes may offer advantages over such enhancers because of their natural origin as we has Generally Regarded As Safe (GRAS) status (Thakur et al., 2006; Dai et al., 2018). Recently, menthol including l-menthol and d-menthol had been selected as a lead compound to synthesize a series of *O*-acylmenthol derivatives which had obvious penetration effects on the drugs both *in vitro* and *in vivo* by our research group (Zhao et al., 2008a,b; Zhao et al., 2009; Wang et al., 2018; Li et al., 2018). Additionally, quantitative structure–activity relationship (QSAR) study has revealed that the activity of terpenes is primarily related to their chemical structures and the unsaturated terpenes seem to be better candidates than the saturated ones in promoting the transdermal absorption (Ghafourian et al., 2004; Kang et al., 2007; Iyer et al., 2007). Since *α*-terpineol (TER) is one kind of cyclic terpenes with a double bond, it has been shown effective in promoting the percutaneous absorption of various drugs with low skin irritation (Fang et al., 2007; Liu et al., 2011; Varman & Singh, 2012; Shi et al., 2013). However, application of TER in transdermal drug delivery systems would be hindered by its undesirable odor and high volatility. Therefore, TER was selected as a leading compound to synthesize *O*-acylterpineol derivatives which have a structure that can be considered to be a chemical combination of TER and fatty acids as candidates for percutaneous absorption enhancers. In order to further evaluate the penetration effects and elucidate the enhancing mechanism of *O*-acylterpineol, known standard enhancers including Azone with a long tail and NMP with a short tail were also employed as reference enhancers in the present study. In general, chemical enhancers were thought to decrease skin barrier resistance by affecting the lipid region of the SC or altering the secondary structures of skin proteins (Patil & Saraogi, 2014; Liu et al., 2017a), However, data concerning the molecular interaction between SC lipids and enhancers, which plays an important role in understanding the mechanisms of enhancement, is still lacking so far.

The purpose of this study was to explore the feasibility of *O*-acylterpineol for use as permeation enhancers, and to investigate their advantages in transdermal enhancement by comparison with Azone and NMP. Isosorbide dinitrate (ISDN) which possessed suitable physicochemical properties for transdermal delivery was selected as a model drug to evaluate the enhancing efficiency of the studied compounds (Zhao et al., 2008a,b). In the present study, with the molecular modeling, confocal laser scanning microscopy (CLSM) and attenuated total reflection Fourier transform infrared spectroscopy (ATR-FTIR) analysis, the alterations in the hydrophobic moieties and polar domain of SC lipid after skin treatment with different enhancers, the regulation and the underlying action mechanisms of these chemicals in the skin microstructures, including the barrier properties of SC and the conformational order of the intercellular skin lipids were respectively investigated. Finally, with *in vitro* skin cells and *in vivo* erythema models, the skin irritation and toxicity of the synthesized chemicals were also evaluated.

## Materials and methods

### Materials

ISDN supplied by Jianglong Pharmaceutical Co, Ltd (Shanxi China). Acetanilide and TER were purchased from Beijing Xingjin Chemical Plant (Beijing, China); isopropyl myristate (IPM), Azone, *N*-methyl-2-pyrrolidone (NMP), propanoic acid, butyric acid, hexanoic acid, heptanoic acid, decanoic acid, dodecanic acid, tetradeconic acid, stearic acid, oleic acid, tetrahydrofuran (THF), and diethyl sulfate were supplied by China National Medicines Co., Ltd (Shanghai, China); Duro-Tak® (DT) adhesives were obtained from National Starch and Chemical Co., USA; Methanol of HPLC grade were obtained from the fisher Co, Ltd (NJ, USA); Fluorescein was provided by AXROS Organics (NJ, USA).

### Synthesis of O-acylterpineol derivatives

The chemical structures and reaction sequences of *O*-acylterpineol derivatives are listed in Supporting Information. The purity of each compound was over 98% as shown by gas chromatography (GC-14C, Shimadzu, Japan). The structures of the compounds were confirmed by NMR (ARX-300, Bruker, Switzerland) and HPLC-MS (ZQ-2000, Waters, USA). The ^1^HNMR and MS data are as follows:

#### 2 -(4-Methylcyclohex-3-en-l-yl) propan-2-yl propionate (TER-C3)

ESI-MS *m/z*: 211.3 [*M* + 1]^+^; ^1^H NMR(CDCl_3_), *δ*: 0.90(3H, s), 1.01(3H, s), 1.0(3H, s), 1.25(3H, s), 1.30–1.35(2H, m), 1.40(1H, m), 1.58–1.60(2H, m), 1.87(1H, m), 2.04(2H, m), 2.22(1H, m), 4.70(1H, d, *J =* 11.2 Hz). Yield: 78%.

#### 2 -(4-Methylcyclohex-3-en-l-yl) propan-2-yl butanoate (TER-C4)

ESI-MS *m/z*: 225.3 [*M* + 1]^+^; ^1^H NMR(CDCl_3_), *δ*: 0.90(3H, s), 1.01(3H, s), 0.99(3H, s), 1.25(3H, s), 1.30–1.35(4H, m), 1.41(1H, m), 1.58–1.60(2H, m), 1.88(1H, m), 2.03(2H, m), 2.20(1H, m), 4.70(1H, d, *J =* 11.2 Hz). Yield: 75%.

#### 2 -(4-Methylcyclohex-3-en-l-yl) propan-2-yl hexanoate (TER-C6)

ESI-MS *m/z*: 253.4 [*M* + 1]^+^; ^1^H NMR(CDCl_3_), *δ*: 0.90(3H, s), 1.00(3H, s), 1.01(3H, s), 1.24 (3H, s), 1.30–1.35(8H, m), 1.40(1H, m), 1.58–1.60(2H, m), 1.89 (1H, m), 2.04 (2H, m), 2.22 (1H, m), 4.70(1H, d, *J =* 11.2 Hz). Yield: 70%.

#### 2 -(4-Methylcyclohex-3-en-l-yl) propan-2-yl heptanoate (TER-C7)

ESI-MS *m/z*: 267.4 [*M* + 1]^+^; ^1^H NMR(CDCl_3_), *δ*: 0.91(3H, s), 0.99(3H, s), 1.01(3H, s), 1.23 (3H, s), 1.30–1.36(10H, m), 1.40(1H, m), 1.58–1.62 (2H, m), 1.89(1H, m), 2.03 (2H, m), 2.21 (1H, m), 4.70(1H, d, *J =* 11.2 Hz). Yield: 71%.

#### 2 -(4-Methylcyclohex-3-en-l-yl) propan-2-yl decanoate (TER-C10)

ESI-MS *m/z*: 309.5 [*M* + 1]^+^; ^1^H NMR(CDCl_3_), *δ*: 0.91(3H, s), 1.00(3H, s), 1.01(3H, s), 1.25–1.37(19H, m), 1.40(1H, m), 1.58–1.61 (2H, m), 1.89(1H, m), 2.04 (2H, m), 2.21 (1H, m), 4.70(1H, d, *J =* 11.2 Hz). Yield: 72%.

#### 2 -(4-Methylcyclohex-3-en-l-yl) propan-2-yl dodecanoate (TER-C12)

ESI-MS *m/z*: 337.5 [*M* + 1]^+^; ^1^H NMR(CDCl_3_), *δ*: 0.90(3H, s), 0.99(3H, s), 1.01(3H, s), 1.25–1.36(23H, m), 1.40(1H, m), 1.57–1.61 (2H, m), 1.89(1H, m), 2.03 (2H, m), 2.21 (1H, m), 4.70(1H, d, *J =* 11.2 Hz). Yield: 67%.

#### 2 -(4-Methylcyclohex-3-en-l-yl) propan-2-yl tetradecanoate (TER-C14)

ESI-MS *m/z*: 365.5 [*M* + 1]^+^, ^1^H NMR(CDCl_3_), *δ*: 0.91(3H, s), 0.99(3H, s), 1.01(3H, s), 1.24–1.36(27H, m), 1.40(1H, m), 1.57–1.61 (2H, m), 1.89(1H, m), 2.03 (2H, m), 2.21 (1H, m), 4.70(1H, d, *J =* 11.2 Hz). Yield: 68%.

#### 2-(4-Methylcyclohex-3-en-l-yl) propan-2-yl stearate (TER-C18)

ESI-MS *m/z:* 421.6 [*M* + 1]^+^; ^1^H NMR(CDCl_3_), *δ*: 0.91(3H, s), 0.99(3H, s), 1.01(3H, s), 1.24–1.42(36H, m), 1.56–1.61 (2H, m), 1.89(1H, m), 2.03 (2H, m), 2.21 (1H, m), 4.70(1H, d, *J =* 11.2 Hz). Yield: 68%.

#### (E)-2-(4-Methylcyclohex-3-en-l-yl) propan-2-yl octadec-9-enoate (TER-Cd18)

ESI-MS *m/z:* 419.6 [*M* + 1]^+^; ^1^H NMR(CDCl_3_), *δ*: 0.91(3H, s), 0.99(3H, s), 1.01(3H, s), 1.24–1.65(34H, m), 1.89(1H, m), 2.03 (2H, m), 2.21 (1H, m), 2.38(2H, t, *J =* 7.4 Hz), 4.70(1H, d, *J =* 11.2 Hz). Yield: 55%.

## Permeation *experiments*

### Preparation of donor solutions

Donor solutions of the ISDN were obtained by equilibration of excess amounts of solute in IPM, with and without selected concentration enhancers, then vortexed for 2 min followed by sonication for 10 min to dissolve the drug. The molar concentration of the enhancer in this study was based on the concentration of *α*-terpineol which was screened (0.32 mmol in 1 g IPM) and an excess amount of solute was present throughout the experiments.

### Preparation of DIA patches containing ISDN

The DIA (drug in adhesive) patches containing 10% ISDN (w/w) were prepared with various PSA (pressure sensitive adhesive) and enhancers, the amount of ISDN was the same for all dosing formulations. A laboratory-coating unit (SLT200, Kaikai Co., Ltd, Shanghai, China) was used to prepare the DIA patches. An appropriate amount of ISDN was dissolved in a suitable amount of ethyl acetate, which was later added to the PSA solution and mixed thoroughly with a mechanical stirrer. The resulting drug-PSA solution was coated onto a fluoropolymer-treated polyester release liner (ScotchPak^®^ 1022, 3M, USA) at a thickness of 80 μm. After the solvent had been removed, it was laminated with a polyester backing film (ScotchPak^®^ 9732, 3M).

### Skin preparation

Male rabbits weighing 1500–1800 g (4–6 months old) used in all experiments were supplied by the Experimental Animal Center of North China University of Science and Technology (Tangshan, China). All the animal procedures were performed in accordance with the NIH Guidelines for the Care and Use of Laboratory Animals and also in accordance with the guidelines for animal use published by the Life Science Research Center of North China University of Science and Technology. The rabbits were anesthetized with urethane (20% w/v, i.p) and the back was carefully shaved with a razor after removal of hair by electric clippers (model 900, TGC, Japan). Full thickness skin (i.e. epidermis with SC and dermis) was excised from the shaved back site. The integrity of the skin was carefully ascertained by microscopic observation, any skin which had low uniformity was rejected. After removing the fat and subdermal tissue, the skin was kept frozen at –20 °C and used within 1 week. Before starting the experiments, the skin was allowed to reach room temperature for at least 10 h (hereafter h).

### *In vitro* permeation experiments

Skin permeation experiments were performed according to the previous report (Zhao et al., 2008a). A diffusion cell consisting of two half-cells with a water jacket connected to a water bath at 32 °C was used. Each half-cell had a volume of 2.5 ml and an effective area of 0.95 cm^2^. The dermis side of the skin was in contact with the receiver compartment and the SC with the donor compartment. The donor compartment was filled with the drug suspension and the receiver compartment with pH 7.4 PBS. During all the experiments, excess drug was maintained in the donor compartment. Both donor and receiver compartments were stirred with a star-head bar driven by a constant speed synchronous motor at 600 rpm. In the patch application study, the dermis side of the skin was attached to one-half of the side-by-side diffusion cells. The ISDN patch was applied to the SC side of the skin, and pH 7.4 PBS was used as the receptor medium. In both application studies 2.0 ml of receiver solution was withdrawn at predetermined intervals for measurement of the permeated drug, and fresh receptor medium was added to maintain a constant volume. The solution in the donor or receiver compartment was stirred with a star-head bar driven by a constant speed synchronous motor at 600 rpm.

### Drug analysis

The HPLC system for analyzing drug concentrations was equipped with a SPD-20A variable-wavelength ultraviolet absorbance detector, a LC-20AT pump and a SIL-20A automatic sampler (Shimadzu High-Technologies Corporation, Tokyo, Japan). Samples were introduced 20 µl into a Rheodyne Model 7725 loop injector equipped with a 20 μl loop. The reversed phase stainless-steel column (15 cm × 4.6 mm) was packed with Diamonsil C-18 (5 µm particle size; Dikma Technologies, Beijing, China). The HPLC conditions of ISDN were as follows: The mobile phase consisted of methanol and 0.1% acetic acid in distilled water (50:50, v/v), the wavelength was set at 230 nm, and acetanilide was used as internal standard.

### Determination of drug solubility

To determine the saturation solubility of the ISDN in IPM, with and without enhancer, excess drug was added to known volumes of vehicle, vortexed for 2 min followed by sonication for 10 min to dissolve the drug and then equilibrated at 32 ± 0.5 °C for more than 48 h. Finally, the contents were centrifuged at 16,000 rpm for 10 min and aliquots for the supernatant saturated solution were diluted and analyzed by HPLC. The experiments were performed in triplicate.

### Data analysis

The cumulative amount of drugs permeating through the skin from IPM system or DIA patch was plotted as a function of time. The skin flux was determined from Fick’s law of diffusion, listed in Equation (1):

(1) $$J_{s}=dQ_{r}/Adt$$

The cumulative amount of drugs permeating through the skin at 8 h (*Q*_8_) or 24 h (*Q*_24_) was calculated from the drug concentration in the receiver compartments. The flux was calculated from the slope of the linear portion of the profiles. The lag-time was determined by extrapolating the linear portion of the curve to the abscissa; where *J*_s_ is the steady-state skin flux in µg/cm^2^/h, *dQ*_r_ is the change in quantity of the drug passing through the skin into the receptor compartment in µg, *A* is the active diffusion area in cm^2^, and *dt* is the change in time, *T*_lag_ is the lag-time in h. The permeability coefficient (*P*) was calculated according previous reports as Equation (2)

(2) $$P=J_{\text{s}}/C_{\text{s}}$$

where *C*_s_ is the saturated solubility of drugs in donor solutions.

To evaluate the promoting activity of each enhancer, enhancement ratios (ER) were calculated as skin parameters (*Q*_8_ or *Q*_24_) for the enhancer-containing group divided by the same parameter for the control (no enhancer present). Controls were assigned a value of 1.00.

## Microdialysis *experiment*

### Microdialysis system

In the present research, the microdialysis method was based on a previous report (Xie et al., 2016). The microdialysis system consisted of an 11-Elite micro-infusion pump (Harvard Apparatus, USA) with a 2.5 ml glass syringe (Hamilton, USA) and a CMA 30 linear microdialysis probe (inner diameter, 0.24 mm; outer diameter, 0.38 mm; molecular cutoff, 6000 Da). The inlet tube of the probe was connected to the microinjection pump using polyethylene tubing. In all the experiments, the length of the membrane accessible to dialysis was 10 mm, and the perfusate flow rate was 1.0 μl/min. Cannulas were used as insertion guides, and vials were used to collect the dialysate samples.

### Recovery validation *in vitro* and correlation *in vivo*

The *in vitro* recovery was assessed by a concentration difference method, which was performed to ensure the retrodialysis method was suitable for this *in vivo* recovery study. A linear microdialysis probe was placed in a 25 ml Franz diffusion cell containing different concentrations (0.5, 1.0, 2.0, 4.0, 8.0, 16.0, 32.0, 64.0 μg/ml^–1^) of ISDN in sequence, and the dialysis membrane portion of the probe was completely immersed in the solution at 32 ± 0.5 °C. The probe was perfused with drug solution (5.0 μg/ml^–1^) at a flow of 1.0 ml/min. After an equilibration period of 1 h, the dialysate was collected into a HPLC vial for another 1 h. Dialysate samples were analyzed using HPLC to determine the drug concentration. *In vitro* relative recovery was calculated as the slope of the linear regression of drug concentration difference between dialysate (*C*_d_) and perfusate (*C*_p_) as a function of drug concentration in the perfusate (*C*_p_). When the drug concentration in dialysate (*C*_d_) is higher than that in perfusate (*C*_p_), the slope of the linear regression actually refers to the delivery of the microdialysis probe, whereas the slope should be the recovery of the probe, this data analysis method was also based on a previous report (Gao et al., 2014).

*In vivo* recovery ratio was determined using the retrodialysis method, which relies on the assumption that net drug transport from the perfusate into the surrounding tissues through the microdialysis membrane is equal to that from the tissues into the perfusate. The diffusive loss of ISDN was determined, and recovery ratio was calculated from the following equation: Recovery (*in vivo*) = (*C*_p_ – *C*_d_)/*C*_p_. To determine the diffusive loss of ISDN, the rabbits were anesthetized with urethane (20% w/v, i.p). The linear probe was inserted into the dermis of the back skin. After perfusion with PBS for 1 h, the standard drug solution was used as perfusate. HPLC assays were conducted to determine the loss of drug from the standard drug solutions.

### *In vivo* microdialysis studies

Prior to administration of ISDN, the rabbits were anesthetized during *in vivo* microdialysis. The back was carefully shaved with a razor after removal of hair by electric clippers in each rabbits. The microdialysis probe was implanted in the dermis, and the active dialysis window was placed immediately below the site of DIA patch administration. The probe was continuously perfused with PBS. The skin was allowed to equilibrate for 1 h before a blank sample was taken from the microdialysis probe and 1.5 h after the start of perfusion. A DIA patch of about 30 mm in diameter was administered above the application site. Dialysate samples were collected into HPLC vials, which were replaced every 1.5 h. Dialysis sampling was continued for 24 h. As with the *in vitro* skin permeation studies, DIA patches with or without enhancers were used to investigate the *in vivo* penetration-enhancing role of the enhancers on ISDN.

### ATR-FTIR experiment

The rabbits were anesthetized with urethane (20%, w/v, i.p) and the back hair was shaved as mentioned above. Then the rabbit back was divided into 12 different sections with each area of 1 cm^2^. 10 μl of each enhancer solution (10 mg dissolved in 100 μl ethanol) was applied onto the marked areas for an exposure time of 30 min. Then the treated skin segments were excised, then the skin was blotted with tissue paper and dried at room temperature for 15 min (*n* = 3), determined by an ATR-FTIR spectroscopy (Thermo Nicolet NEXUS 470, Madison, USA), the treated skin was placed on the surface of crystal with the SC side down. The internal reflection crystal was zinc selenide (ZnSe) set at an incidence angle of 45°. The spectra were obtained as an average of 200 scans recorded between 4000 cm^–1^ and 1000 cm^−1^ at a resolution of 2 cm^−1^. Location of the IR absorbance-peak maximum was determined to an accuracy of 0.1 cm^−1^ using Origin 7.0 (software). The spectrum of the skin treated only with 100 μl ethanol was also recorded as control group.

### Molecular modeling

Eight-times repeat units of ceramide NP (Cer NP) were built in three-dimensional (3D) coordinates by means of Sybyl 6.91 software package (Tripos, Inc., ST. Louis, MO, USA). After addition of all the hydrogen atoms with Amber 7 FF99 force field, partial charges were assigned to Cer NP molecules according to the method of Gastei-ger-Hückel. The structures were subjected to further energy minimization using both steepest descent and conjugate gradient protocols.

Docking calculations were preformed on Auto Dock 3.05 (the Scripps Reaearch Institute, La Jolla, FL, USA). The fully flexible ISDN, TER, TER-C4, TER-C14, NMP, and Azone, were built in 3D coordinates consistently with the method for the construction of Cer NP assemblies. Biopolymer module was then used to proceed with flexible torsions in the ligands and therefore all dihedral angles were allowed to rotate freely. The docking procedure was levied on the whole CER receptor without imposing the binding site (“blind docking”). A 60 × 60 × 60 grid box with a grid spacing of 0.375 Å was generated, which was large enough to totally cover the overall surface of the receptor. Affinity grid fields were obtained from the auxillary program AutoGrid 3.0. Lamarckian genetic algorithm (LGA) was probed to find the most favorable complex geometry, i.e. the most favorable interactions. Optimized AutoDocking parameters were stated as follows: for LGA searching algorithms the maximum number of energy evaluations was increased to 25,000,000 per run; the iterations of Solis & Wets local search were 3000; the number of individuals in population was set to 300; and the number of generations was set to 100. All other parameters were set to the default settings. Cluster analysis of AutoDock results was carried out to determine whether different binding sites have generated from multiple runs (Morris et al., 1998;Chen et al., 2013).

### Confocal laser scanning microscopy (CLSM)

According to previous report (Chen et al., 2014), saturated fluorescein solution was used in the CLSM analysis. As described in preparation of donor vehicles, the enhancer containing IPM solutions were first prepared. An excess amount of fluorescein was then added to the formulation, followed by sonication for 10 min. The suspensions were centrifuged at 5000 rpm for 5 min, and the supernatants were subsequently filtered through a 0.45 μm millipore filter. The control sample was obtained in a similar way without addition of any enhancer.

Full thickness rabbit skin was mounted in the Franz diffusion cells. The donor cells were placed with 200 μl fluorescein solution, while the receptor cells were filled with PBS. After skin exposure for 20 min, the excess solution was removed from the skin surface, and then the diffusion cells were dismantled. The SC surface of the skin was thoroughly wiped with distilled water before drying gently with filter paper. The skin segment was immediately sandwiched between a glass slide and a coverslip, and examined microscopically without additional tissue processing.

An LSM 710 Laser Scan Microscope (Carl Zeiss Carl Zeiss, Jena, Germany) was used for imaging of the skin samples. The fluorescein was excited using an argon laser at a wavelength of 488 nm. *Z* stacks of the skin samples were taken from the SC to the dermis. To visualize the distribution of fluorescein, confocal images were first obtained in the *xy* plane (i.e. parallel to the plane of the skin surface). The skin surface (*z* = 0 μm) was defined as the imaging plane of florescence with a morphology characteristic of the SC surface. To generate an *xz* section, a horizontal line was “drawn” across a region of interest in the *z* = 0 μm *xy* plane and then “optically sliced” through the digitized image data of the successive *xy* sections to generate *xz* planar optical cross-sections (Alvarez-Román et al., 2004).

### *In vitro* skin cells cytotoxicity assays

The keratinocytes and fibroblasts in the viable skin layers play important roles in the initiation, modulation, and regulation of skin inflammation. The present cytotoxicity studies were performed on human skin fibroblasts (HSF) or keratinocytes (HaCaT) cell lines using the 3-(4,5-dimethylthiazol-2-yl)-2,5-diphenyltetrazolium bromide (MTT, Sigma, USA) assay. Cell viability was determined after 24 h treatment with different permeation enhancers at various concentrations (0–3 mmol/L) in 96-well plates. The relative amount of viable cells was determined by measuring the reduction of MTT dye in live cells to blue formazan crystals at optical density at 570 nm and expressed as the percentage of solvent control samples without enhancer treatment.

### *In vivo* skin erythema analysis

Visual observation of erythema has been widely used to evaluate the irritant potential of substances. However, this method is subjective and imprecise, especially regarding comparisons between the intensities of developed Erythema (Nose & Tsurumi, 1993). A noninvasive *in vivo* skin erythema analysis was conducted to monitor the irritation of the enhancers. The erythema index of the skin was tested by Mexameter^®^ (MX 16; Courage & Khazaka Co., Germany). Before the treatment of the enhancers, the initial erythema index was determined and labeled as EI_0_. The EIt was recorded at predetermined time and ΔEI was used to evaluate the skin irritation of the enhancers. EI was calculated as Equation (3):

(3) $$\Delta {\text{EI=EI}}_{\text{t}}{\text{/EI}}_{\text{0}}$$

A 10% (w/v) aqueous solution of sodium dodecyl sulfate (SDS) was used as a positive control in each rabbit. TER, TER-C14, and 10% SDS of 500 μl were respectively applied on the shaved skin, which was then covered with a sheet of double-layer gauze to prevent any skin damage. After exposure of 4 h, the excess solution was removed and the application site was gently cleaned with a water soaked cotton wool swab. At different intervals, EIt was determined automatically, the experiments were performed in quadruplicates.

### Statistical analysis

All parameters were reported as the mean ± SE. Statistical analysis was carried out using analysis of variance (ANOVA). The level of significance was taken as *p* < .05. A correlation analysis was performed with the help of the SPSS program, and correlation coefficients were examined for significance (*p* < .05) using Student’s *t*-test.

## Results and discussion

### In vitropermeation study

#### Percutaneous of ISDN with different α-terpineol concentrations *in vitro*

When cumulative amounts permeating as a function of the enhancer concentration were compared at 8 h, a wide fluctuation was apparent, shown in Supporting Information. At 0%, 1%, 3%, 5%, and 10% TER level (w/w), the cumulative transport of ISDN was 536.41 ± 15.60 μg/cm^2^, 462.29 ± 73.98 μg/cm^2^, 941.32 ± 93.35 μg/cm^2^, 1574.32 ± 70.62 μg/cm^2^, and 793.78 ± 105.25 μg/cm^2^. It can be seen that at 5% concentration, the cumulative permeation of ISDN was higher than the other levels (*p* < .05), while there was no significant difference between 3% and the 10% group (*p* > .05), so the 5% level was chosen to perform latter experiment. The concentration of *O*-acylterpineol derivatives during the subsequent experiments were at the same molar concentration with TER which was selected for ISDN.

#### Percutaneous absorption of ISDN *in vitro*

The effects of *O*-acylterpineol, NMP, and Azone on the percutaneous permeation parameters of ISDN (solubility, flux, *T*_lag_, *P, Q*_8_, and ER) through rabbit skin are shown in Table 1. All the enhancers had obviously enhancing effects on the percutaneous permeation of ISDN relative to the control (*p* < .05). TER-C4 and NMP increased the ISDN in *Q*_8_ by 7.67-fold and 8.50-fold relative to the control, respectively, followed by TER-C6 (6.35-fold), TER-C14 (5.31-fold), TER-C12 (4.75-fold), and TER-C3 (4.26-fold). While no significant difference was found among TER-C16, TER-C7, TER-C18, and Azone (*p* > .05), their cumulative amounts are almost identical (*p* > .05).

**Table 1.** Permeation parameters of ISDN through rabbit skin.

Enhancer	ISDN					
	Solubility (μg/ml)	Flux (μg/cm^2^/h)	*T*_lag_ (h)	*P* (cm/h) × 10^3^	*Q*_8_ (μg/cm^2^)	ER
Control	10,188.26 ± 1126.71	78.42 ± 4.11	1.48	6.60 ± 0.92	536.41 ± 15.60	1.00
5%-Terpineol	21,267.72 ± 483.62	266.82 ± 38.76*	1.95	12.55 ± 1.82*	1574.32 ± 70.62*	2.94
TER-C3	18,824.21 ± 2077.68	349.22 ± 59.31*	1.65	18.54 ± 3.15*	2285.10 ± 424.68*	4.26
TER-C4	16,714.44 ± 636.28	639.33 ± 49.35**	1.51	39.53 ± 3.05**	4141.59 ± 312.86**	7.67
TER-C6	27,177.80 ± 1254.66	522.75 ± 60.12**	1.28	19.24 ± 0.2.21*	3406.38 ± 320.92**	6.35
TER-C7	25368.31 ± 1326.16	294.80 ± 22.5*	1.73	11.62 ± 0.88*	1856.89 ± 122.22*	3.45
TER-C10	23,933.31 ± 712.17	302.84 ± 21.17*	1.10	12.65 ± 0.88*	2065.88 ± 134.30*	3.85
TER-C12	24,975.81 ± 963.54	392.75 ± 70.76**	1.42	15.72 ± 2.83*	2547.14 ± 411.89**	4.75
TER-C14	23,722.31 ± 1167.24	473.50 ± 49.84**	1.66	19.96 ± 0.2.10*	2848.15 ± 323.10**	5.31
TER-C18	24,523.60 ± 1606.83	243.25 ± 16.39*	1.88	10.21 ± 0.69*	1527.52 ± 122.54*	2.85
TER-Cd18	25,611.23 ± 622.35	333.28 ± 46.27*	1.81	13.01 ± 1.81*	1881.80 ± 214.26*	3.50
NMP	24,738.77 ± 1056.64	787.67 ± 61.36**	2.01	31.84 ± 2.48**	4561.77 ± 313.28**	8.50
Azone	24,592.21 ± 985.33	251.75 ± 17.55*	1.52	10.08 ± 10.31*	1806.10 ± 124.68*	3.37

The receiver phase used an identical (pH 7.4 PBS) and donor phases consisted of IPM; IPM: terpineol (20:1) (w/w), and equivalent molar *O*-acylterpineol with terpineol in IPM. Data are given as average ± SE (*n* = 4). ER is the enhancement ratio calculated as follows: ER = *Q* (with enhancer)/*Q* (without enhancer).

*Significantly different from control group, *p* < .05.

**Significantly different from terpineol group, *p* < .05.

The present research revealed that TER-C4 with an cyclic double bond at C (1) had a significantly higher enhancement activity than M-BUT which was synthesized by l-menthol and butyric acid on the transdermal absorption of ISDN (Zhao et al., 2008a). These results agreed well with previous reports that the unsaturated terpenes seem to be better candidates than the saturated ones in promoting the transdermal absorption (Ghafourian et al., 2004; Kang et al., 2007; Iyer et al., 2007). In the case of *O*-acylterpineol, which had different promoting activity towards the transdermal penetration of ISDN, despite them with the same polar head. Their enhancing activity was strongly associated with the tail lengths. Therefore, a mechanism investigation at a molecular level was suggested to understand the acting mechanisms for TER, *O*-acylterpineol derivatives and other enhancers employed in the research.

### Effect of PSAs and O-acylterpineol on skin permeation of ISDN

The selection of an appropriate PSA is the most important factor in designing a TDDS (Tan & Pfister 1999). For the formulation of a DIA patch containing ISDN, the effect of different types of PSA on the skin permeation of ISDN was evaluated using excised rabbit skin. The ISDN concentration in the PSAs was fixed at 10% (w/w) and each DIA patch was prepared with a thickness of 80 μm. Of the PSAs chosen as matrix, DT-2287 showed the highest skin permeation rate of ISDN, shown in Table 2. Therefore, DT-2287 was selected as the PSA of choice for the DIA patch containing ISDN. Since TER-C4, TER-C6, TER-C10, TER-C12, TER-C14, NMP produced high skin permeation rates of ISDN in IPM solution, their enhancing effects on the skin permeation of ISDN from DIA patches were also investigated, and the results are demonstrated in Table 2. The skin permeation rate of ISDN with the *α*-terpineol group was almost the same as with the control. TER-C14 produced the highest increase in *Q* (543.15 ± 23.10 μg/cm^2^) (*p* < .05). However, unlike solution formulations, the addition of TER-C4 and NMP failed to produce the highest *Q* value.

**Table 2.** Skin permeation rates of ISDN through excised rabbit skin from the patches containing 10% ISDN (w/w) and different enhancers in PSAs.

Trade name^a^	Enhancers	Flux(μg/cm^2^/h)	*Q*_24_ (μg/cm^2^)	*T*_lag_ (h)	ER^b^
DT4098	Control	11.64 ± 1.77	165.80 ± 14.26	7.17	–
DT9301	Control	8.53 ± 1.56	102.77 ± 13.28	6.54	–
DT2287	Control	15.33 ± 1.33	235.10 ± 24.68	6.98	1.00
	TER	12.68 ± 1.53	241.59 ± 12.86	5.13	1.02
	TER-C4	22.31 ± 2.24*	446.38 ± 20.92*	4.87	1.90
	TER-C6	22.28. ± 2.95*	406.89 ± 22.22*	5.13	1.73
	TER-C10	26.29 ± 5.33*	380.88 ± 34.30*	5.17	1.62
	TER-C12	24.36 ± 3.24*	416.14 ± 41.89*	4.27	1.77
	TER-C14	28.48 ± 3.51*	543.15 ± 23.10*	3.13	2.31
	NMP	19.04 ± 1.95*	410.52 ± 22.54*	4.83	1.75

Data are given as average ± SE (*n* = 4).

^a^Duro-Tak series made by Nation Starch and Chemical Co., USA.

^b^ER is the enhancement ratio calculated as follows: ER = *Q* (with enhancer)/*Q* (without enhancer).

*Value is significantly different from ISDN in control (*p* < .05).

As drug transdermal permeation in patches consists of two processes, the first is drug release from patches and the second is its percutaneous absorption. The present result suggests that the permeation rate of ISDN may depend not only on the promoting effect of enhancer, but also on the release speed of the drug from the patch. The potential interaction between the –NO_2_ of ISDN and the –CO– (carbonyl) of DT-2287 may play a negative role in the release and diffusion of the ISDN from the patch, as the lag time is nearly 7 h of control patch. Recently, some interesting reports have been published by researchers who proposed that chemical enhancers could decrease drug-adhesive interaction by the formation of free volume of adhesive, which facilitated drug release process (Song et al., 2016; Liu et al., 2016, 2017a). In the present research, by comparing the effects of chemical enhancers on lag time, TER-C14 was supposed to influence ISDN release process more significantly, thereby providing higher accumulation than other enhancers. However, more detailed investigation is required to further elucidate the mechanisms of interaction among the ISDN, *O*-acylterpineol, and DT-2287.

### *In vivo* skin microdialysis

From the *in vitro* recovery validation studies, a linear relationship of ISDN was found between *C*_p_ and *C*_d_ – *C*_p_ (*C*_d_ – *C*_p_ = –0.7782*C*_p_ + 1.3524, *r* ^2^ = 0.9991). The results implied that no interaction between the ISDN and dialysis membrane occurred in PBS solution, and the ISDN transport from the perfusate into the surrounding tissues through the dialysis membrane was equal to that from the tissues into the perfusate. Results from the *in vitro* studies had shown that the microdialysis probe was not the rate-limiting factor for drug diffusion in the dialysis, the *in vivo* recovery by retrodialysis could be used to correct the data from *in vivo* microdialysis samples. According to the *in vivo* recovery studies using the retrodialysis method, the *in vivo* recovery of ISDN kept a stable value of 41.35 ± 1.77% in 24 h.

It has been shown theoretically that “hydrophilic” drugs will be enhanced most by agents that have a positive impact on the SC diffusion process. Conversely, “lipophilic” drugs will be transported more efficiently if the enhancer can, in some way, facilitate the SC-viable tissue partitioning step (Wenkers & Lippold, 1999). As the model drug ISDN with the balance of lipophilicity and hydrophilicity (log *P* = 1.34) (Zhao et al., 2008a) was suitable for investigating the *in vivo* penetration-enhancing role of *O*-acylterpineol, so the *in vivo* recovery for ISDN was relatively high and stable.

**Figure 1.**
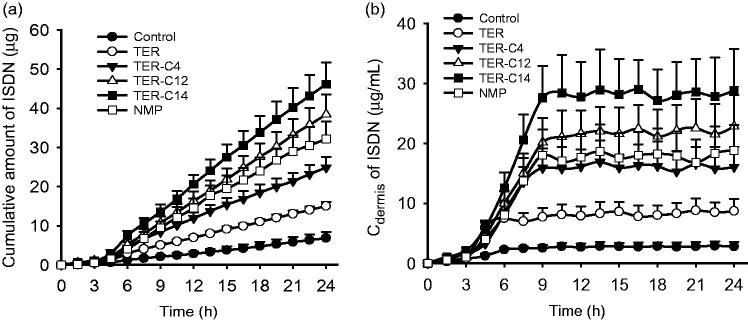
*In vivo* transdermal permeation profiles of ISDN after treated with DIA patches with different enhancers. (a) The cumulative amount profiles of ISDN; (b) the drug concentration–time curves of ISDN collected from the dermis. (*n* = 6).

The drug concentration–time curves and cumulative amount profiles of ISDN in dialysate collected from the dermis with or without enhancers were displayed in Figure 1. Similar to the *in vitro* skin permeation studies, *O*-acylterpineol significantly promoted the *in vivo* transdermal flux or cumulative amounts of ISDN in comparison with the control, and 5% TER-C14 also exhibited the highest efficiency compared with other enhancers. Despite the fact that *in vitro* and *in vivo* skin penetration studies exhibited different transdermal parameters due to the difference between *in vitro* and *in vivo* environment, these enhancers exhibited a similar penetration behavior on the transdermal absorption of ISDN, which further verified the results of the *in vitro* skin penetration studies.

### ATR-FTIR experiments

The ATR-FTIR was selected to provide information of the lipid in the surface layer of the skin, a blue shift in these peaks indicates that the vibration and disorder of the hydrocarbon chains might occur (Obata et al., 2010; Li et al., 2017). The present experiments of ATR-FTIR were carried out with TER, TER-C4, TER-C14, and NMP, respectively. As ethanol alone is inefficient to produce any changes in the C–H stretching absorbance from SC lipids (Bhatia et al., 1997; Zhao & Singh, 1998) and the adequacy of providing rapid reversibility of its action would be expected after application on the skin (Ibrahim & Li, 2009). Consequently, exposure of the rabbit skin to ethanol would not affect the determination of ATR-FTIR.

**Figure 2.**
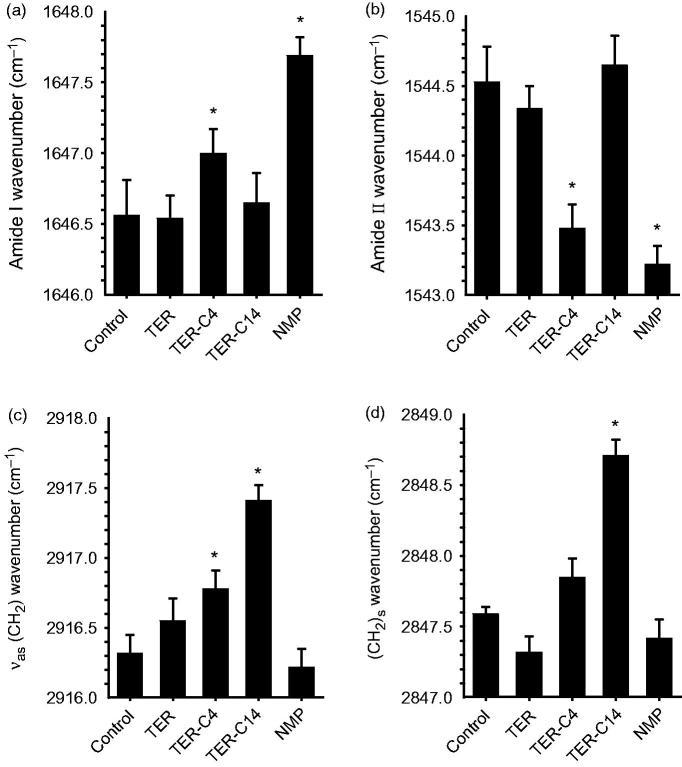
The effects of enhancers on the protein (panels a and b) and lipid (panels c and d) components of SC studied using IR spectroscopy. (a) and (b) Wavenumbers of amide I bands at around 1650 cm^−1^ and amide II bands at around 1540 cm^−1^, respectively; (c) and (d) wavenumbers of asymmetric and symmetric methylene stretching of SC lipids. Mean ± SE, *n* ≥ 4; *Statistically significant difference compared to the respective control at *p* < .05.

The results (outlined in Figure 2) indicated that the rabbit skin treated with TER-C14 produced a higher shift in asymmetric/symmetric C–H vibration peak positions (around 2920 and 2850 cm^−1^), compared with control, however, neither TER nor NMP produced any blue or red shift in these stretching peak positions. Both TER-C4 and NMP produced a higher shift in the stretching vibration peak position of amide I (carbonyl stretch νC = O in the CO–NH group, around 1650 cm^−1^) and a lower shift the stretching vibration peak position of amide II (plane bending mode νNH, around 1540 cm^−1^), while there were no obvious peak shifts of amide I and amide II after the enhancer treatment by TER and TER-C14 compared to control.

Casal and Mantsch have explained the shift in C–H stretching absorption at a molecular level (Casal & Mantsch, 1984). The shift to higher frequency occurred when CH_2_ groups along the alkyl chain of lipids changed from trans to gauche conformation, and indicated that the SC lipid was perturbed. The lipids are thus thought to exist in a more fluid-like state and this is termed the liquid crystalline phase. The magnitude of the shift in C–H stretching vibration was directly related to the ratio of trans to gauche conformers in the alkyl chain. The higher the shifts, the higher the ratio of trans to gauche. It was demonstrated that the promoters causing the higher shift of C–H stretching vibration improved drug permeation (Levang et al., 1999; Takahashi et al., 2001). It can be concluded that the ability of TER-C14 to disorder the SC lipids was more potent than that of TER-C4 and TER. However, the TET-C4 which had higher penetration effect than TET-C14 produced lower spectral shift of C–H stretching vibration than that of the TET-C14. This result suggested that, besides the enhancement mechanism of perturbing the SC lipids, another mechanism plays an important role in penetration of TER-C4.

Comparing TER-C14 with control, the amide II bands shifted reproducibly by about 1.2 cm^−1^ to lower wavenumbers after application of TET-C4, the similar effect was also observed after application NMP. A previous literature report states that the observed amide II bands in SC at about 1540 and 1508 cm^−1^ can be attributed to α-helical conformation and β-pleated sheets, respectively (Lin et al., 1996). Recently, a lot of new insights about the protein structure have been proposed by Goormaghtigh’group (Grimard et al., 2004; Sarroukh et al., 2013; Baldassarre et al., 2015; De Meutter et al., 2017), who reported that the tertiary structure of protein also existed and could change in the absence of significant secondary structure modification. The changes could be monitored by kinetics of deuteration or polarized IR light.

In the present investigation, all the changes of wavenumbers observed are less than 2 cm^–1^, which was inconsistent with conversion of the protein from an α-helical conformation to β-pleated sheets that has been reported (Grimard et al., 2004; Sarroukh et al., 2013). So, TER-C4 may penetrate into the corneocytes and disrupt the keratin filament network, thereby rendering the corneocytes more permeable. Therefore, the changes outlined above suggest that increased transdermal flux of ISDN by enhancers correlates with increased SC lipid fluidization and alters the SC protein interaction caused by exposure to TET-C4. The promoting activities of TET-C14 and NMP only correlate with SC lipid fluidization or alter the SC protein interaction alone.

It is noteworthy that although TER did not produce any significant change in the peak positions of asymmetric/symmetric C–H and peak position of amide I/II, which showed obvious enhancement effect on the permeation of ISDN. Similar results were also found in previous studies for other terpenes such as menthone and isopulegol (Krishnaiah et al., 2002; Chen et al., 2013). The effective promoting activity of TER for ISDN may be attributable to its “terpene pooling effect.” Cornwell et al. reported that the amount of terpenes taken up by the SC ranged from 8.9% to 39% (Cornwell et al., 1996), Cal et al. also reported that both epidermis and dermis can absorb terpenes (Cal et al., 2001), while the higher affinity of terpenes for epidermis can be demonstrated, and the amount of terpenes found in the epidermis is over 50% of the total mass, which suggests that terpene enhancer pooling may occur in the SC and epidermis. In this study, the TER may pool in SC and epiderm to facilitate the permeation of ISDN. To prove this hypothesis requires further investigation.

### Molecular modeling

Molecular modeling was used to explore the interaction between chemical enhancers and the ceramides which are considered as the essential components for maintaining the skin barrier function in the lipids of SC. Among them, ceramide NP (Cer NP, shown in Supporting Information), equivalent to human ceramide 3, was often used to investigate the effect of penetration enhancers on the skin lipids due to its important role in the lipid organization of SC barrier. In the present study, one Cer NP was first selected as a repeated unit and then eight Cer NP molecules were optimized together to produce favorable polymer chain assemblies of Cer NP (shown in Supporting Information), which was used to simulate a microenvironment of the ceramides in SC. Then the drug or enhancer was added into the system separately and the conformation continued to be optimized until an appropriate one was produced.

**Figure 3.**
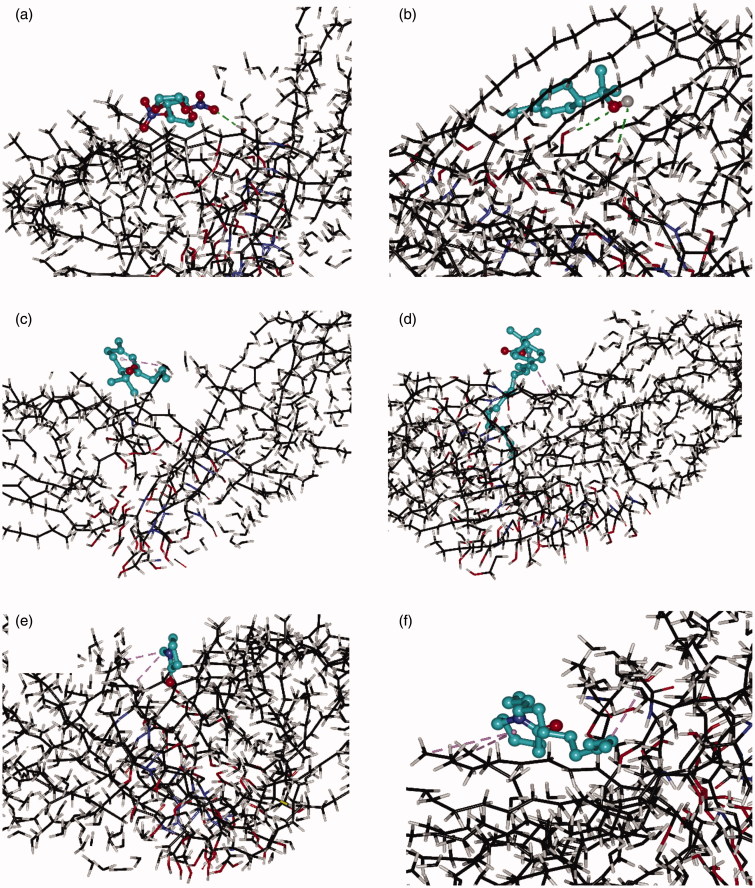
Interaction of the Cer NP assemblies with molecular models of ISDN (a), TER (b), TER-C4 (c), TER-C14 (d), NMP (e), and Azone (f). Carbon atoms were colored black, oxygen atoms red, nitrogen atoms blue, hydrogen atoms gray. These figures are screenshots of the Cer NP assemblies. H-bonds were presented in green dotted lines, hydrophobic interactions were presented in brown dotted lines.

As is shown in Figure 3(a), ISDN partitioned into the ceramides via H-bonds, indicating that it had good affinity with polar domains in the SC (red color containing area). However, different associating sites could be observed for different enhancers, which were shown in Figure 3(b–f). TER located at polar domain (red color containing area) and formed H-bonds with the polar headgroups of the Cer NP assemblies; the other compounds located at lipophilicity domain (black color containing area) and formed hydrophobic interactions with the lipophilicity chains of the Cer NP assemblies. Based on the optimal conformation, the docked energy for ISDN, TER, TER-C4, TER-C14, NMP, and Azone was –3.23 kcal/mol, –4.13 kcal/mol, –4.87 kcal/mol, –5.89 kcal/mol, –4.53 kcal/mol, –9.70 kcal/mol, respectively. The values were generally negative, suggesting that the interaction of Cer NP assemblies with the drug or enhancers was possible. The intercellular spaces of SC contain structured lipids and a diffusing molecule has to cross a variety of hydrophobic and polar domains before it reaches the viable epidermis (Cal et al., 2001). Previous research reported that there was a possibility that the occupation of terpenes in the polar lipid domain could disturb the interaction between the 5-fluorouracil and ceramides, pulling the drug free of H-bonds and increasing the diffusivity across SC (Krishnaiah et al., 2002). In the case of ISDN and TER, which formed H-bonds with the polar headgroups of the Cer NP assemblies, the lower docked energy was provided by TER, potentially indicative that it had the greater affinity to the polar groups of the ceramides than ISDN. It could preferentially interact with the polar group of the lipids, which offered a reasonable explanation for its enhancement activity.

As far as the other enhancers (TER-C4, TER-C14, NMP, and Azone) were concerned, which formed hydrophobic interactions with the lipophilicity chains of the Cer NP assemblies, the experimentally determined ER decreased with increasing the absolute values of docked energy. As docked energy increases, the enhancer should bind more tightly with the SC, and the amount of free enhancer which can facilitate the penetration of ISDN decreased to give an inverse relationship between ER and docked energy. In the present research, we can speculate that for enhancers conjunction with SC by hydrophobic interactions, the higher absolute values in the docked energy between compound and SC, the weaker promoting activities that can be obtained in IPM donor solution for permeant partitioned into the polar domain of ceramides via H-bonds. Certainly to prove this hypothesis requires further investigation.

### CLSM analysis

**Figure 4.**
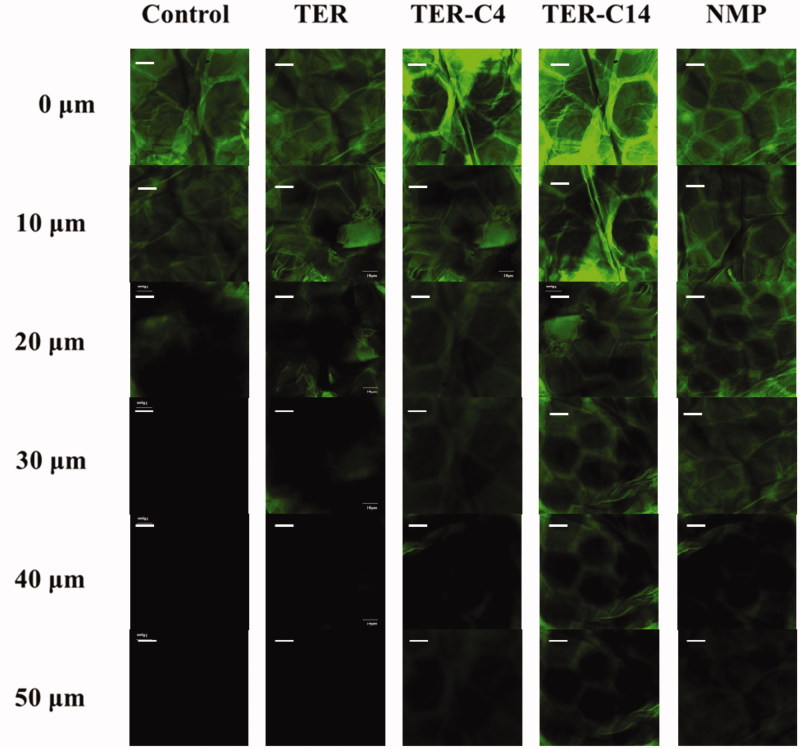
CLSM images of the rabbit skin after the treatment by IPM solution for 20 min with or without penetration modifiers. (a) Solvent blank, (b) TER, (c) TER-C4, (d) TER-C14, and (e) INMP. Scale bar represents 10 μm. (*n* = 4).

CLSM was used to visualize the penetration activity of *O*-acylterpineol derivatives. As ISDN could not be detected by CLSM, fluorescein was used instead in this study for its greater molecular weight compared with that of ISDN (MW = 236.14). The greater MW of fluorescein may imply that it would be more difficult to penetrate across the rabbit skin, which is of vital importance to reflect the transdermal process of ISDN. There have been many literature works which examined permeation characteristics of different enhancers by using fluorescein as a fluorescent probe (Chen et al., 2014; Li et al., 2017; Zhao et al., 2017). In the present research, authors examined the rabbit skin samples subsequent to the application of (a) solvent blank, (b) TER, (c)TER-C4, (d) TER-C14, and (e) NMP. The *xz* optical sections of the skin after treatment with fluorescein solutions in different groups are shown in Figure 4. The morphology of the surface layers identically confirmed with the reported “brick and mortar” of the SC, and the flat hexagonal structure of the horny cells was clearly observed at the skin surface (Obata et al., 2010; Chen et al., 2014). In specific, in the control group, it was shown that fluorescein could penetrate into the skin up to 20 μm without addition of any permeation modifiers (Figure 4(a)). When the skin was pretreated with enhancers, significant increase on the penetration depth was observed, indicating that TER, TER-C4, TER-C14, and NMP showed enhancement effect on the skin penetration of fluorescein (Figure 4(b–e)). In TER group, the fluorescein probe was observed at the depth of 20 μm of the skin, while, the penetration depth of fluorescein reached 30 μm, 40 μm, 40 μm and 50 μm for TER, TER-C4, NMP, and TER-C14, respectively. These results were consistent with that of the *in vitro* and *in vivo* penetration study. CLSM was utilized to visualize the permeation of exogenous molecule in the skin under the influence of the permeation modifiers. According to the result of CLSM, in the group of TER-C14, the florescence probe was clearly observed beyond the epidermis and into the dermal layer, which indicated that TER-C14 could effectively breach the barrier function of the epidermis and increase the skin permeability, as the thickness of rabbit epidermis providing an efficient permeation obstacle for exogenous molecules was less than 40 μm (Godin & Touitou, 2007).

### *In vitro* cytotoxicity (MTT assay) and *in vivo* skin irritation

Though most transdermal penetration enhancers showed fairly satisfactory performance in facilitating the permeation of drug molecule across the skin, few of them have been approved for clinical use on account of their skin irritation. The epidermal keratinocytes and dermal fibroblasts were employed to gain insights into the cytotoxicity of *O*-acylterpineol and roughly evaluated its skin irritation. The cell viabilities of keratinocytes and fibroblasts after treatment with various concentrations of the two compounds are shown in Supporting Information. Following 24 h incubation, TER and TER-C14 showed dose-dependent cytotoxicity on the keratinocytes and fibroblasts. However, both of them did not induce a 50% reduction in the cells viability up to the high dosage of 3 mmol/l. No significant difference was observed between the cytotoxicity of TER and TER-C14, however, the trend of decrease in the cytotoxicity to both of the two cell lines by TER-C14 was indicated in comparison with TER.

As TER-C14 was derived from natural compound (TER), it was expected to possess low irritancy potential. After treatment of SDS, TER, and TER-C14, the skin erythema was significantly increased, suggesting that the rabbit could respond adequately to the skin irritants, illustrated in Supporting Information. The greatest skin color change was provided by SDS, and this effect did not decline at all throughout the experiment. There was no significant difference between TER and TER-C14, and about 24 h was required for the enhancer treated skin to achieve the complete skin recovery. The irritation experiments demonstrated that TER and TER-C14, could both slightly increase the skin erythema, but their action would be easily reversible.

## Conclusion

From the results of this investigation, it is concluded that some newly designed percutaneous absorption enhancers were shown to be promising candidates as permeation enhancers for clinical transdermal drug delivery, with the advantages of high efficiency, low skin irritation, and toxicity. The structure–activity relationships of *O*-acylterpineol derivatives are summarized as follows: tail chain length has important effects on the enhancing activity, C14 seems to be more favorable for the penetration of ISDN from DIA patches. The mechanistic insights studies suggested that *O*-acylterpineol could increase lipid fluidization and alter the protein conformation of SC. Meanwhile, the *O*-acylterpineol with long chain (C14) could penetrate into the dermal layer of the rabbit skin. These reports also represented some new ideas for the penetration enhancer design and extended the understanding about the acting mechanisms for enhancers.

## Supplementary Material

xin_dd_Supplementary-xiu.docx

## References

[CIT0001] Alvarez-Román R, Naik A, Kalia YK, et al. (2004). Enhancement of topical delivery from biodegradable nanoparticles. Pharm Res 21:1818–25. 15553228 10.1023/b:pham.0000045235.86197.ef

[CIT0002] Baldassarre M, Li C, Eremina N, et al. (2015). Simultaneous fitting of absorption spectra and their second derivatives for an improved analysis of protein infrared spectra. Molecules 20:12599–622. 26184143 10.3390/molecules200712599 PMC6331840

[CIT0003] Bhatia KS, Gao S, Singh J (1997). Effect of penetration enhancers and iontophoresis on the FT-IR spectroscopy and LHRH permeability through porcine skin. J Control Release 47:81–9. 10.1021/js970023k 9294814

[CIT0004] Cal K, Janicki S, Sznitowska M (2001). In vitro studies on penetration of terpenes from matrix-type transdermal systems through human skin. Int J Pharm 224:81–8. 11472817 10.1016/s0378-5173(01)00744-x

[CIT0005] Casal HL, Mantsch HH (1984). Polymorphic phase behaviour of phospholipid membranes studied by infrared spectroscopy. Biochim Biophys Acta 779:381–401. 6391546 10.1016/0304-4157(84)90017-0

[CIT0006] Chen Y, Cun D, Quan P, et al. (2014). Saturated long-chain esters of isopulegol as novel permeation enhancers for transdermal drug delivery. Pharm Res 31:1907–18. 24449443 10.1007/s11095-013-1292-0

[CIT0007] Chen Y, Wang J, Cun D, et al. (2013). Effect of unsaturated menthol analogues on the in vitro penetration of 5-fluorouracil through rat skin. Int J Pharm 443:120–7. 23333756 10.1016/j.ijpharm.2013.01.015

[CIT0008] Cornwell PA, Barry BW, Bouwstra JA, et al. (1996). Modes of action of terpene penetration enhancers in human skin, differential scanning calorimetry, small-angle X-ray diffraction and enhancer uptake studies. Int J Pharm 127:9–26.

[CIT0009] Dai X, Wang R, Wu Z, et al. (2018). Permeation-enhancing effects and mechanisms of borneol and menthol on Ligustrazine: a multiscale study using in vitro and coarse-grained molecular dynamics simulation methods. Chem Biol Drug Des 92:1830–7. 29923687 10.1111/cbdd.13350

[CIT0010] De Meutter J, Vandenameele J, Matagne A, et al. (2017). Infrared imaging of high density protein arrays. Analyst 142:1371–80. 27924984 10.1039/c6an02048h

[CIT0011] Fang JY, Tsai TH, Lin YY, et al. (2007). Transdermal delivery of tea catechins and theophylline enhanced by terpenes: a mechanistic study. Biol Pharm Bull 30:343–9. 17268077 10.1248/bpb.30.343

[CIT0012] Gao Q, Zhao Y, Yu J, et al. (2014). Microdialysis as a tool to determine the skin concentration of mometason furoate in rats. Pharmazie 69:787–91. 25985571

[CIT0013] Ghafourian T, Zandasrar P, Hamishekar H, et al. (2004). The effect of penetration enhancers on drug delivery through skin: a QSAR study. J Control Release 99:113–25. 15342185 10.1016/j.jconrel.2004.06.010

[CIT0014] Godin B, Touitou E (2007). Transdermal skin delivery: predictions for humans from in vivo, ex vivo and animal models. Adv Drug Deliv Rev 59:1152–61. 17889400 10.1016/j.addr.2007.07.004

[CIT0015] Grimard V, Li C, Ramjeesingh M, et al. (2004). Phosphorylation-induced conformational changes of cystic fibrosis transmembrane conductance regulator monitored by attenuated total reflection-Fourier transform IR spectroscopy and fluorescence spectroscopy. J Biol Chem 279:5528–36. 14660584 10.1074/jbc.M311014200

[CIT0016] Hadgraft J (1999). Passive enhancement strategies in topical and transdermal drug delivery. Int J Pharm 184:1–6. 10425346 10.1016/s0378-5173(99)00095-2

[CIT0017] Hui X, Wester RC, Zhai H, et al. (2005). Chemical partitioning into powdered human stratum corneum: a useful in vitro model for studying interaction of chemicals and human skin In: Bronaugh RL, Maibach HI, eds. Percutaneous absorption. Boca Raton (FL): Taylor & Francis,291–302.

[CIT0018] Ibrahim SA, Li SK (2009). Effects of solvent deposited enhancers on transdermal permeation and their relationship with Emax. J Control Release 136:117–24. 19331847 10.1016/j.jconrel.2009.01.023 PMC2823392

[CIT0019] Iyer M, Zheng T, Hopfinger AJ, et al. (2007). QSAR analyses of skin penetration enhancers. J Chem Inf Model 47:1130–49. 17472334 10.1021/ci700051e

[CIT0020] Kang L, Yap WC, Lim CFP, et al. (2007). Formulation development of transdermal dosage forms-quantitative structure–activity relationship model for predicting activities of terpenes that enhance drug penetration through human skin. J Control Release 120:211–19. 17582639 10.1016/j.jconrel.2007.05.006

[CIT0021] Krishnaiah YSR, Satyanarayana V, Karthikeyan RS (2002). Permeation enhancing effect of menthol on the percutaneous flux of nicardipine hydrochloride through excised rat epidermis from hydroxypropyl cellulose gels. Pharm Dev Technol 7:305–15. 12229262 10.1081/pdt-120005727

[CIT0022] Levang AK, Zhao K, Singh J (1999). Effect of ethanol/propylene glycol on the in vitro percutaneous absorption of aspirin, biophysical changes and the macroscopic barrier properties of the skin. Int J Pharm 181:255–63. 10370221 10.1016/s0378-5173(99)00055-1

[CIT0023] Li N, Quan P, Wan X, et al. (2017). Mechanistic insights of the enhancement effect of sorbitan monooleate on olanzapine transdermal patch both in release and percutaneous absorption processes. Eur J Pharm Sci 107:138–47. 28693956 10.1016/j.ejps.2017.07.006

[CIT0024] Li Y, Zhao L, Liu C (2018). Effect of chiral *O*-acylmenthol derivatives on in vitro permeation of flubiprofen. Chin J Pharm 49:962–7.

[CIT0025] Lin SY, Duan KJ, Lin TC (1996). Microscopic FT-IR/DSC system used to simultaneously investigate the conversion process of protein structure in porcine stratum corneum after pretreatment with skin penetration enhancers. Methods Find Exp Clin Pharmacol 18:175–81. 8738068

[CIT0026] Liu C, Quan P, Fang L (2016). Effect of drug physicochemical properties on drug release and their relationship with drug skin permeation behaviors in hydroxyl pressure sensitive adhesive. Eur J Pharm Sci 93:437–46. 27575879 10.1016/j.ejps.2016.08.048

[CIT0027] Liu C, Quan P, Li S, et al. (2017a). A systemic evaluation of drug in acrylic pressure sensitive adhesive patch in vitro and in vivo: the roles of intermolecular interaction and adhesive mobility variation in drug controlled release. J Control Release 252:83–94. 28274741 10.1016/j.jconrel.2017.03.003

[CIT0028] Liu CH, Chang FY, Hung DK (2011). Terpene microemulsions for transdermal curcumin delivery: effects of terpenes and cosurfactants. Colloids Surf B Biointerfaces 82:63–70. 20828994 10.1016/j.colsurfb.2010.08.018

[CIT0029] Liu X, Quan P, Li S, et al. (2017b). Time dependence of the enhancement effect of chemical enhancers: molecular mechanisms of enhancing kinetics. J Control Release 248:33–44. 28088574 10.1016/j.jconrel.2017.01.017

[CIT0030] Liu X, Liu M, Liu C, et al. (2017c). An insight into the molecular mechanism of the temporary enhancement effect of isopulegol decanoate on the skin. Int J Pharm 529:161–7. 28610893 10.1016/j.ijpharm.2017.06.023

[CIT0031] Marwah H, Garg T, Goyal AK, et al. (2016). Permeation enhancer strategies in transdermal drug delivery. Drug Deliv 23:1–15. 25006687 10.3109/10717544.2014.935532

[CIT0032] Morris GM, Goodsell DS, Halliday RS, et al. (1998). Automated docking using a Lamarckian genetic algorithm and an empirical binding free energy function. J Comput Chem 19:1639–62.

[CIT0033] Nose T, Tsurumi K (1993). Pharmacological studies on cutaneous inflammation induced by ultraviolet irradiation (1): quantification of erythema by reflectance colorimetry and correlation with cutaneous blood flow. Jpn J Pharmacol 62:245–56. 8411774 10.1254/jjp.62.245

[CIT0034] Obata Y, Utsumi S, Watanabe H, et al. (2010). Infrared spectroscopic study of lipid interaction in stratum corneum treated with transdermal absorption enhancers. Int J Pharm 389:18–23. 20079819 10.1016/j.ijpharm.2010.01.007

[CIT0035] Patil UK, Saraogi R (2014). Natural products as potential drug permeation enhancer in transdermal drug delivery system. Arch Dermatol Res 306:419–26. 24481830 10.1007/s00403-014-1445-y

[CIT0036] Sarroukh R, Goormaghtigh E, Ruysschaert JM, et al. (2013). ATR-FTIR: a "rejuvenated" tool to investigate amyloid proteins. Biochim Biophys Acta 1828:2328–38. 23746423 10.1016/j.bbamem.2013.04.012

[CIT0037] Shi J, Cong W, Wang Y, et al. (2013). Synergistic effect and mechanism of cineole and terpineol on in-vitro transdermal delivery of huperzine A from microemulsions . Iran J Pharm Res 12:271–80. 24250600 PMC3813235

[CIT0038] Song W, Quan P, Li S, et al. (2016). Probing the role of chemical enhancers in facilitating drug release from patches: mechanistic insights based on FT-IR spectroscopy, molecularmodeling and thermal analysis. J Control Release 227:13–22. 26896738 10.1016/j.jconrel.2016.02.027

[CIT0039] Takahashi K, Sakano H, Yoshida M, et al. (2001). Characterization of the influence of polyol fatty acid esters on the permeation of diclofenac through rat skin. J Control Release 73:351–8. 11516511 10.1016/s0168-3659(01)00359-5

[CIT0040] Tan HS, Pfister WR (1999). Pressure-sensitive adhesives for transdermal drug delivery systems. Pharm Sci Technol Today 2:60–9. 10234208 10.1016/s1461-5347(99)00119-4

[CIT0041] Thakur RA, Wang Y, Michniak BB, (2006). Essential oils and terpenes In: Smith EW, Maibach HI, eds. Percutaneous penetration enhancers. Boca Raton (FL): CRC Press,159–73.

[CIT0043] Varman RM, Singh S (2012). Investigation of effects of terpene skin penetration enhancers on stability and biological activity of lysozyme. AAPS PharmSciTech 13:1084–90. 22930344 10.1208/s12249-012-9840-1 PMC3513477

[CIT0044] Wang T, Wang C, Li Y, et al. (2018). Effect of chiral isopropyl O-acylmenthol derivatives on in vitro permeation of metoprolol. J Shenyang Pharm Univ 35:163–9.

[CIT0045] Wenkers BP, Lippold BC (1999). Skin penetration of nonsteroidal antiinflammatory drugs out of a lipophilic vehicle: influence of the viable epidermis. J Pharm Sci 88:1326–31. 10585230 10.1021/js990032o

[CIT0046] Williams AC, Barry BW (2004). Penetration enhancers. Adv Drug Deliv Rev 56:603–18. 15019749 10.1016/j.addr.2003.10.025

[CIT0047] Kadhum WR, Sekiguchi S, Hijikuro I, et al. (2017). Esters of 6-aminohexanoic acid as skin permeation enhancers: transdermal delivery of atorvastatin calcium from novel nanovesicular systems using polyethylene glycol fatty acid esters: ameliorated effect without liver toxicity in poloxamer 407-induced hyperlipidemic rats. J Control Release 254:10–22. 28344015 10.1016/j.jconrel.2017.03.039

[CIT0048] Xie F, Chai JK, Hu Q, et al. (2016). Transdermal permeation of drugs with differing lipophilicity: effect of penetration enhancer camphor. Int J Pharm 507:90–101. 27154251 10.1016/j.ijpharm.2016.05.004

[CIT0049] Zhao H, Liu C, Quan P, et al. (2017). Mechanism study on ion-pair complexes controlling skin permeability: effect of ion-pair dissociation in the viable epidermis on transdermal permeation of bisoprolol. Int J Pharm 532:29–36. 28830782 10.1016/j.ijpharm.2017.08.080

[CIT0050] Zhao K, Singh J (1998). Mechanisms of percutaneous absorption of tamoxifen by terpenes: eugenol, d-limonene and menthone. J Control Release 55:253–60. 9795076 10.1016/s0168-3659(98)00053-4

[CIT0051] Zhao L, Fang L, Xu Y, et al. (2008a). Transdermal delivery of penetrants with differing lipophilicities using O-acylmenthol derivatives as penetration enhancers. Eur J Pharm Biopharm 69:199–213. 18065216 10.1016/j.ejpb.2007.10.015

[CIT0052] Zhao L, Fang L, Xu Y, et al. (2008b). Effect of O-acylmenthol on transdermal delivery of drugs with different lipophilicity. Int J Pharm 352:92–103. 18053661 10.1016/j.ijpharm.2007.10.017

[CIT0053] Zhao L, Li Y, Fang L, et al. (2009). Transdermal delivery of tolterodine by O-acylmenthol: in vitro/in vivo correlation. Int J Pharm 374:73–81. 19446762 10.1016/j.ijpharm.2009.03.005

