# Supplementary material for: Permeation-enhancing effects and mechanisms of O-acylterpineol on isosorbide dinitrate: mechanistic insights based on ATR-FTIR spectroscopy, molecular modeling, and CLSM images
Source: Drug Deliv. 2019 Feb 11;26(1):107–19. doi: 10.1080/10717544.2018.1561764 (PMC6374923; doi:10.1080/10717544.2018.1561764)
Supplement: xin_dd_Supplementary-xiu.docx [file IDRD_A_1561764_SM5162.docx]

**Figure 1.** The chemical structure of *O*-acylterpineol derivatives used as percutaneous absorption enhancers.

**Figure 2.** The reaction sequences for the preparation of *O*-acylterpineol derivatives.

**Figuire.3.** Permeation profiles of ISDN through rabbit skin (average±SE, n=4).


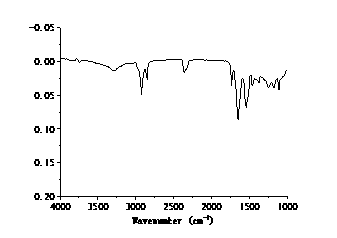

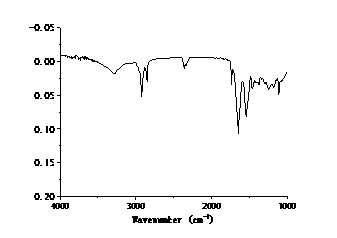


(a) (b)


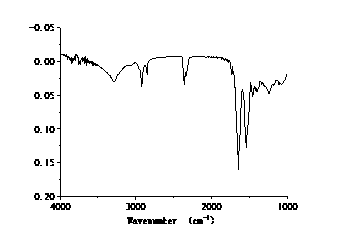

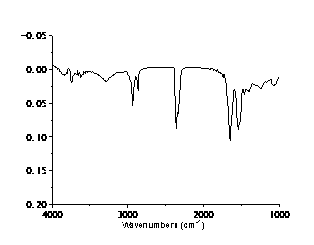


(c) (d)


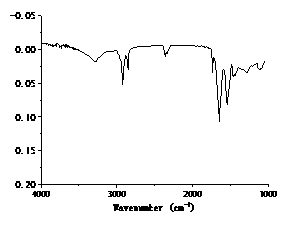


(e)

**Figure 4.** ATR-FTIR spectra of the rabbit skin after the treatment by different enhancers. (a) Solvent blank, (b) TER, (c) TER-C4, (d) NMP, and (e) TER-C14.

**(b)**


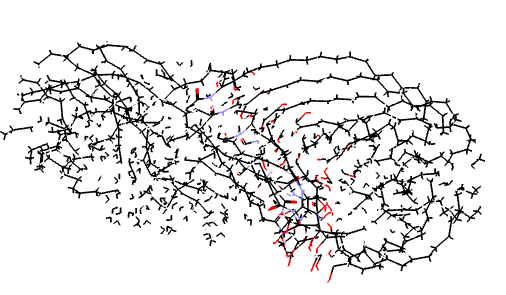


**Figure 5.** Chemical structures of Cer NP (a) and the polymer chain assemblies of Cer NP (b).

**Figure 6.** Cell viability of keratinocyte (a) and fibroblast (b) after treatment of TER and TER-C14 at different concentrations. Data are presented as themean±S.E. (n=4).

**Figure 7** ΔEI values of the rabbit skin after the treatment of TER, TER-C14 and SDS. Data are presented as the mean ± S.E. (n=4).

Table 1. *In vivo* permeation parameters of ISDN after treated with the patches containing

10% ISDN (w/w) and different enhancers

| Trade name | Enhancers | *J*_ss_ (μg/cm^2^/h) | *Q*_24_ (μg/cm^2^) | T_lag_ (h) | ER ^a^ |
| --- | --- | --- | --- | --- | --- |
| DT2287 | Control | 0.26±0.06 | 6.51±1.53 | 5.11 | 1.00 |
|  | TER | 0.62±0.06* | 14.27±0.96* | 4.22 | 2.18 |
|  | TER-C4 | 1.02±0.12* | 23.61±2.54* | 4.07 | 3.62 |
|  | TER-C12 | 1.51±0.22* | 36.66±4.81* | 3.97 | 4.69 |
|  | TER-C14 | 1.86±0.22* | 43.82±5.21* | 3.54 | 6.74 |
|  | NMP | 1.31±0.21* | 30.31±4.24* | 3.73 | 4.67 |

Data are given as average ± S.E. (n=6).

^a^ ER is the enhancement ratio calculated as follows: ER = *Q* (with enhancer)/*Q* (without enhancer).

* Value is significantly different from ISDN in control (*P*<0.05)
